# Fabrication of AlGaN High Frequency Bulk Acoustic Resonator by Reactive RF Magnetron Co-sputtering System

**DOI:** 10.3390/ma14237377

**Published:** 2021-12-01

**Authors:** Yu-Chen Chang, Ying-Chung Chen, Chien-Chuan Cheng

**Affiliations:** 1 Department of Electrical Engineering, National Sun Yat-sen University, Kaohsiung 80424, Taiwan; d043010004@student.nsysu.edu.tw (Y.-C.C.); ycc@mail.ee.nsysu.edu.tw (Y.-C.C.); 2 Department of Electronic Engineering, HungKuo Delin University of Technology, New Taipei 236, Taiwan

**Keywords:** piezoelectric, aluminum gallium nitride, solidly mounted resonator, frequency response

## Abstract

In this study, aluminum gallium nitride (AlGaN) thin films are used as the piezoelectric layers to fabricate solidly mounted resonators (SMR) for high frequency acoustic wave devices. AlGaN film is deposited on a Bragg reflector, composed of three pairs of Mo and SiO_2_ films, through a reactive radio frequency (RF) magnetron co-sputtering system at room temperature. The optimized deposition parameters of AlGaN film have a sputtering power of 175 W for Al target, sputtering power of 25 W for GaN target, N_2_ flow ratio (N_2_/Ar + N_2_) of 60%, and sputtering pressure of 10 mTorr. The obtained AlGaN film has a smooth surface, uniform crystal grains, and strong c-axis orientation. The contents of Al and Ga in the AlGaN film, analyzed by energy dispersive X-ray spectroscopy (EDS) are 81% and 19%, respectively. Finally, the frequency response *s*_11_ of the obtained SMR device shows that the center frequency is 3.60 GHz, the return loss is about −8.62 dB, the electromechanical coupling coefficient (k_t_^2^) is 2.33%, the quality factor (Q) value is 96.93 and the figure of merit (FoM) value is 2.26.

## 1. Introduction

In recent years, wireless communication systems have been advanced to 4G and 5G. At the same time, the acoustic wave devices used in wireless communication equipment have higher requirements, such as frequency response, k_t_^2^, Q, and FoM [1,2,3]. The acoustic wave devices commonly used in wireless communication can be divided into bulk acoustic wave (BAW) and surface acoustic wave (SAW). The bulk acoustic wave device has lower insertion loss, higher frequency, and wider frequency band [4,5,6,7,8]; therefore, it is suitable for high frequency wireless communication system applications. The BAW resonator has two kinds of structure, one is the film bulk acoustic resonator (FBAR), and the other is solidly mounted resonator (SMR). The SMR structure does not require an etching process like the FBAR structure; therefore, it is relatively stable, and the process yield of the device can be improved [8,9,10,11,12,13].

On the other hand, the commonly used piezoelectric materials in acoustic wave devices are aluminum nitride (AlN) and zinc oxide (ZnO) [14,15,16,17]. In recent years, some literature has studied the applications of AlGaN and AlScN in acoustic wave devices [18,19]. Both Wurtzite AlN and GaN have c-axis-oriented polarities and similar piezoelectric characteristics [20]. Therefore, GaN and AlGaN are also suitable for the applications of acoustic wave devices. In order to obtain high qualities of GaN and AlGaN, it often needs high temperature processes [21,22,23]. In 2016, J.B. Shealy et al. successfully fabricated thin film bulk acoustic resonators (FBAR) with single crystal AlGaN thin films, prepared using metal–organic chemical vapor deposition (MOCVD) method [18]. On the other hand, sputtering has lots of advantages such as low temperature growth, enhanced film quality with smaller grain size, and surface roughness, etc. In 2021, N.A. Othman et al. deposited AlGaN thin films on Si substrates through a co-sputtering technique. The structures and morphological characteristics of AlGaN films were discussed [24]. In this study, piezoelectric AlGaN thin films were deposited by a reactive RF magnetron co-sputtering system at room temperature to fabricate solidly mounted resonators (SMR) with a center frequency of around 3.5 GHz.

## 2. Materials and Methods

**Figure 1:**
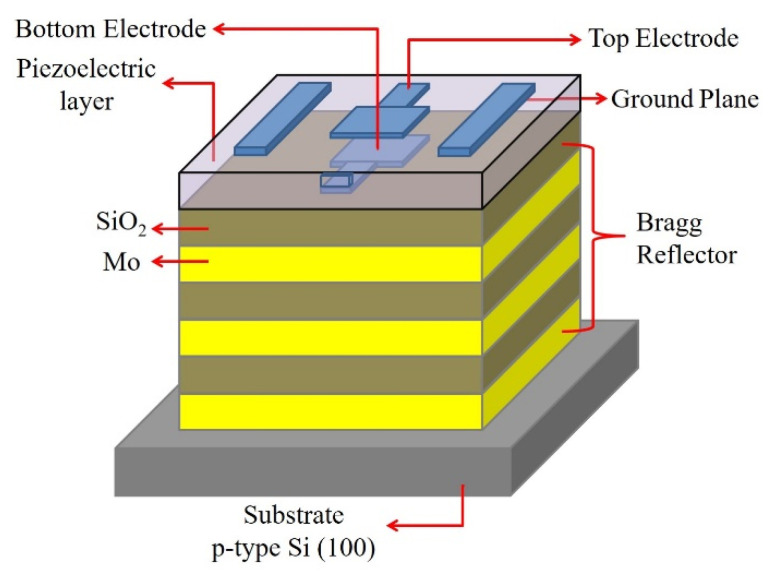
The structure of an SMR device.

The structure of an SMR device, consisting of a Bragg reflector on a Si substrate, a bottom electrode, a piezoelectric layer, and a top electrode, is shown in Figure 1. The Bragg reflector was composed of 3 pairs of Mo and SiO_2_ films as high and low acoustic impedance layers, which were deposited using a direct current (DC) sputtering system and a reactive RF magnetron sputtering system, respectively. The thicknesses of Mo and SiO_2_ films were calculated to be 437 nm and 361 nm to fit the desired 3.5 GHz frequency response of SMR devices, through the theoretical formula of v = f × 4d, in which, v and 4d were the velocity and thickness of individual Mo or SiO_2_ film, respectively, and f was the center frequency of resonance. Pt/Ti (100 nm/12 nm) thin films as the bottom and top electrodes were deposited on the Bragg reflector and piezoelectric layer by a DC sputtering system. The piezoelectric AlGaN thin film was deposited by a reactive RF magnetron co-sputtering system. A 2 inch metallic Al (99.999% purity) and a 2 inch alloyed GaN (99.999% purity) were used as the source targets. The RF sputtering power supplies were applied to Al and GaN targets, separately, and the sputtering pressure was varied from 10 mTorr to 30 mTorr with gas flow ratio (N_2_/N_2_ + Ar) of 60%, to deposit AlGaN thin films at room temperature. The preferred orientation and crystal characteristics of AlGaN films were analyzed by X-ray diffraction (XRD, Bruker D8 Advance) with CuKα radiation. The surface morphologies and cross-sectional images of the AlGaN films were analyzed by a scanning electron microscope (SEM, JEOL JSM-6700F). The element analysis of AlGaN film was carried out by energy dispersive X-ray spectroscopy (EDS, JEOL JSM-6700F). The frequency response of the SMR device was measured by a network analyzer, E5071C, and a CASCADE high-frequency probe station.

## 3. Results and Discussion

In the study of AlGaN thin films, the deposition parameters of sputtering power, sputtering pressure, and gas flow ratio (N_2_/N_2_ + Ar) were varied. The preliminary results showed that films exhibited better characteristics as the sputtering power of 175 W for Al target and 25 W for GaN target, respectively, and the gas flow ratio (N_2_/N_2_ + Ar) of 60% at room temperature. This study was focused on the effects of sputtering pressure on the characteristics of AlGaN thin films. The sputtering pressure varied from 10 to 30 mTorr, and the crystalline structures, the surface morphologies, and the cross-sectional images of AlGaN thin films were discussed.

**Figure 2:**
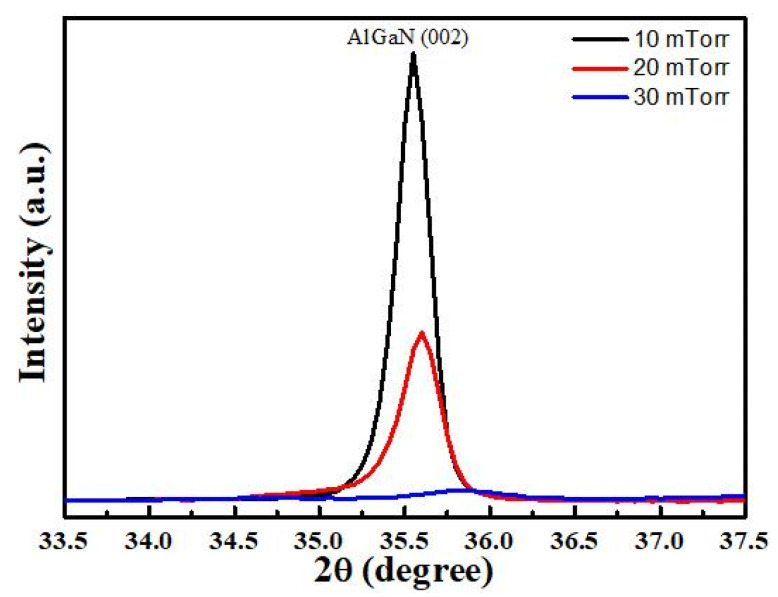
XRD patterns of the AlGaN films deposited under different sputtering pressures.

The XRD patterns of the resulting AlGaN films deposited under different sputtering pressures are shown in Figure 2. It can be seen that the AlGaN films exhibit c-axis (002) orientation under sputtering pressures of 10 mTorr and 20 mTorr. However, as the pressure increases further, the (002) peak intensity of the AlGaN film becomes weaker and even absent in the c-axis orientation. This phenomenon is similar to that reported by N.A. Othman et al. [24]; that is, the crystal quality of AlGaN film decreases as N_2_ flow increases.

According to the literature, the diffraction peak of AlN (002) is about 2θ = 36° [17], while that of the AlGaN film deposited at sputtering pressure of 10 mTorr is about 2θ = 35.55°. There exists a shift of 0.45° for (002) diffraction peak between AlGaN and AlN films. The reason is that when gallium is doped into AlN, part of the larger Ga atoms replaces Al atoms in the lattice, which will result in the change of the lattice structure and cause the shift in the diffraction peak [19,25]. This phenomenon is similar to the doping of Sc atoms into AlN to form ScAlN films [26].

**Figure 3:**
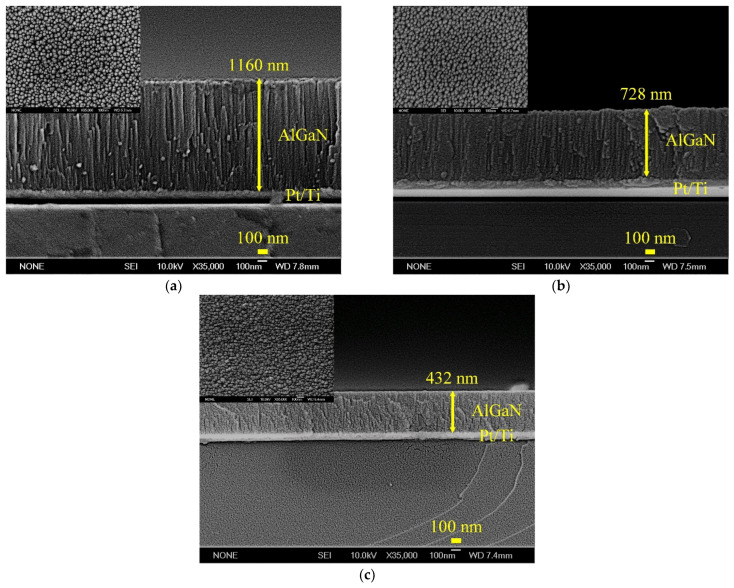
The surface morphologies and cross-sectional images of AlGaN films deposited under different sputtering pressures; (**a**) 10 mTorr, (**b**) 20 mTorr, and (**c**) 30 mTorr.

**Figure 4:**
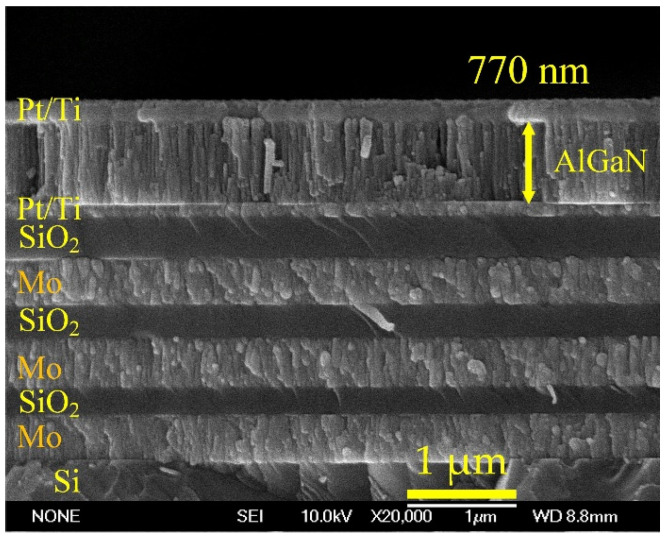
The cross-sectional image of an AlGaN-based SMR device.

The surface morphologies and cross-sectional images of AlGaN thin films deposited under different sputtering pressures were analyzed by SEM, as shown in Figure 3. The films all present a uniform pebble-like surface morphology. However, it can be found from the cross-sectional images that when the sputtering pressure increases, the thickness of AlGaN film decreases from 1160 nm to 432 nm under a deposition time of 3 h, and the film is unlikely to be deposited and there is no columnar structure. As the pressure increases, the structure of the AlGaN film gradually changes from the original pebble-like structure and columnar structure to an agglomerated structure. The overall crystal characteristics are also greatly degraded, which will have a large impact on the fabrication of SMR devices and may make the SMR devices have no frequency response. In order to obtain a 3.5 GHz SMR resonator, three pairs of Mo and SiO_2_ films were deposited on a Si substrate as the Bragg reflector, on which bottom electrode (Pt/Ti), piezoelectric AlGaN layer, and top electrode (Pt/Ti) were deposited in sequence. The cross-sectional image of an AlGaN-based SMR device is shown in Figure 4. The interfaces between the electrode layers, the piezoelectric layers, and the Bragg reflector layers are smooth, uniform, and clearly visible in the fabricated SMR device. The prepared piezoelectric AlGaN film has a columnar structure and c-axis orientation. The thickness of AlGaN film was adjusted to be about 770 nm by controlling the deposition time to fit the desired resonance frequency, according to the theoretical calculation of v = f × 2d—in which v was the wave velocity of AlGaN film, f was the center frequency, and 2d was the thickness of AlGaN film. Besides, the element analysis of energy dispersive X-ray spectroscopy (EDS) showed that the contents of Al and Ga in the AlGaN film were 81% and 19%, respectively.

**Figure 5:**
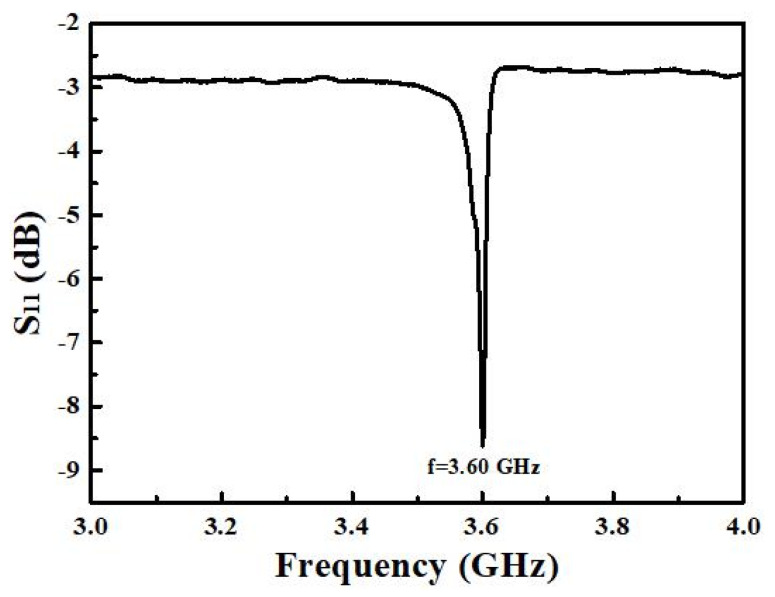
Frequency response of the SMR device with 770 nm AlGaN film as the piezoelectric layer.

The frequency response (*s*_11_) of the SMR device with 770 nm AlGaN film as the piezoelectric layer is shown in Figure 5. The results show that the resonance frequency and return loss of the SMR device are 3.6 GHz and −8.62 dB, respectively.

The frequency responses, k_t_^2^, Q, and FoM of the SMR device can be analyzed through the network analyzer, which are calculated as follows [1,2,3]:

(1) $$k_{t}{}^{2}=\frac{\emptyset }{\tan\emptyset }=\frac{\left(\frac{\pi }{2}\right)\left(\frac{f_{s}}{f_{p}}\right)}{\tan\left(\left(\frac{\pi }{2}\right)\left(\frac{f_{s}}{f_{p}}\right)\right)}≅{\left(\frac{\pi }{2}\right)}^{2}\left(\frac{f_{p}-f_{s}}{f_{p}}\right)$$

(2) $$Q=2\pi f\times \tau \left(f\right)\times \frac{\mathrm{mag}\left(s_{11}\right)}{1-\mathrm{mag}{\left(s_{11}\right)}^{2}}$$

(3) $$\mathrm{FoM}=Q_{\left(\mathrm{device}\right)}\times k_{t}{{}^{2}}_{\left(\mathrm{device}\right)}$$

Among them, f_s_ is the series resonance frequency, f_p_ is the parallel resonance frequency, τ(f) is the time delay of the network analyzer, and mag(*s*_11_) is the return loss (*s*_11_) value. The resulted performance parameters of the SMR device in this work showed that k_t_^2^ was 2.33%, Q value was 96.93, and FoM was 2.26, respectively. By comparing, the return loss, k_t_^2^, Q, and FoM values are worse than those obtained in device using the single crystal AlGaN film as the piezoelectric layer deposited by MOCVD method [19]. The reason may be that the higher the resonance frequency of acoustic wave device, the thinner the piezoelectric layer that is needed, which may result in the poor crystalline characteristics of the piezoelectric layer [8]. The performance of the SMR device can be improved through a thermal annealing process of piezoelectric layer and the precise control of the thicknesses of reflective layers in the Bragg reflector [8].

## 4. Conclusions

In this study, the Bragg reflector, composed of three pairs of Mo and SiO_2_ films, was firstly fabricated on a Si substrate, then the AlGaN thin film was successfully deposited on the Bragg reflector by a reactive RF magnetron co-sputtering system at room temperature. Under a sputtering power of 175 W for Al target, a sputtering power of 25 W for GaN target, a N_2_ flow ratio of 60%, and a sputtering pressure of 10 mTorr, the optimized AlGaN film with a smooth surface, uniform crystal grains, and strong c-axis-orientated crystallization was obtained. The contents of Al and Ga in the AlGaN film were 81% and 19%, respectively.

The AlGaN-based SMR device with good performance was successfully fabricated. The frequency response (*s*_11_) of the SMR device showed that the resonance frequency was 3.60 GHz, the return loss was about −8.62 dB, the k_t_^2^ was 2.33%, the Q value was 96.93, and the FoM value was 2.26, respectively.

## Data Availability

Not applicable.
